# Distinct *Streptococcus pneumoniae* cause invasive disease in Papua New Guinea

**DOI:** 10.1099/mgen.0.000835

**Published:** 2022-07-11

**Authors:** Kate C. Mellor, Stephanie Lo, Mition Yoannes, Audrey Michael†, Tilda Orami, Andrew R. Greenhill, Robert F. Breiman, Paulina Hawkins, Lesley McGee, Stephen D. Bentley, Rebecca L. Ford, Deborah Lehmann

**Affiliations:** ^1^​ Parasites and Microbes, Wellcome Sanger Institute, Hinxton, UK; ^2^​ Papua New Guinea Institute of Medical Research, Goroka, Papua New Guinea; ^3^​ Institute of Innovation, Science and Sustainability, Federation University Australia, Churchill, Australia; ^4^​ Rollins School of Public Health Emory University, Atlanta, GA, USA; ^5^​ Centers for Disease Control and Prevention, Atlanta, GA, USA; ^6^​ Wesfarmers Centre of Vaccines and Infectious Diseases, Telethon Kids Institute, The University of Western Australia, Crawley WA 6009, Australia

**Keywords:** *Streptococcus pneumoniae*, Papua New Guinea, invasive disease

## Abstract

*
Streptococcus pneumoniae* is a key contributor to childhood morbidity and mortality in Papua New Guinea (PNG). For the first time, whole genome sequencing of 174 isolates has enabled detailed characterisation of diverse *S. pneumoniae* causing invasive disease in young children in PNG, 1989-2014. This study captures the baseline *S. pneumoniae* population prior to the introduction of 13-valent pneumococcal conjugate vaccine (PCV13) into the national childhood immunisation programme in 2014. Relationships amongst lineages, serotypes and antimicrobial resistance traits were characterised, and the population was viewed in the context of a global collection of isolates. The analyses highlighted adiverse *S. pneumoniae* population associated with invasive disease in PNG, with 45 unique Global Pneumococcal Sequence Clusters (GPSCs) observed amongst the 174 isolates reflecting multiple lineages observed in PNG that have not been identified in other geographic locations. The majority of isolates were from children with meningitis, of which 52% (*n*=72) expressed non-PCV13 serotypes. Over a third of isolates were predicted to be resistant to at least one antimicrobial. PCV13 serotype isolates had 10.1 times the odds of being multidrug-resistant (MDR) compared to non-vaccine serotype isolates, and no isolates with GPSCs unique to PNG were MDR. Serotype 2 was the most commonly identified serotype; we identified a highly clonal cluster of serotype 2 isolates unique to PNG, and a distinct second cluster indicative of long-distance transmission. Ongoing surveillance, including whole-genome sequencing, is needed to ascertain the impact of the national PCV13 programme upon the *S. pneumoniae* population, including serotype replacement and antimicrobial resistance traits.

## Data Summary

Genome sequences were deposited in the European Nucleotide Archive (ENA), the accession number and the sample metadata are available in the supplementary material. The authors confirm all supporting data, code and protocols have been provided within the article or through supplementary data files.

**Impact Statement**

*Streptococcus pneumoniae*is a major cause of bacteraemic pneumonia and bacterial meningitis in Papua New Guinea (PNG). A 13-valent pneumococcal conjugate vaccine (PCV13) was introduced into the national immunisation programme in 2014. This study analyses samples prior to vaccine introduction, and finds a diverse *S. pneumoniae* population with evidence of the maintenance of multiple lineages that were distinct from those identified in other countries. Concordant with published literature, the results indicate that the majority of cases of invasive pneumococcal disease, in particular meningitis, in PNG are caused by non-PCV13 serotypes, limiting its potential benefit in PNG; however higher valency vaccines in development may be more effective. Over a third of pneumococcal isolates were resistant to one or more antimicrobials and 7.3 % of isolates were multidrug resistant (MDR). Vaccine serotype isolates were 10.1 times more likely to be MDR than non-vaccine serotype isolates which suggests potential reduction in AMR with use of PCV13. However, continued monitoring is needed to assess replacement of dominant *S. pneumoniae* serotypes and lineages in PNG following the perturbation caused by introduction of PCV13.

## Background

*Streptococcus pneumoniae* is highly endemic and a common cause of pneumonia and meningitis in Papua New Guinea (PNG) [1–3]. Papua New Guinea encompasses the eastern half of the island of New Guinea and its offshore islands. Neighbouring countries include Australia, Indonesia and the Solomon Islands. The predominantly rural population of ~9.1 million people [4] live in highland, coastal and island regions and speak >800 different languages [5]. The mortality rate in children <5 years-of-age is high (57 per 1000 births in 2015) and pneumonia is the most common cause of hospitalisation and death in this age group [6, 7]. Chloramphenicol was the first-line treatment for children with meningitis, but has now been replaced by ceftriaxone due to an increase in cases of meningitis caused by chloramphenicol resistant *Haemophilus influenzae* [8, 9]. Intermediate penicillin resistance of *S. pneumoniae* has long been reported in PNG [10, 11] and was detected in 21.5% of pneumococcal isolates from cerebrospinal fluid (CSF) in children between 1996 and 2005 [2]. A recent increase in resistance of *S. pneumoniae* to cotrimoxazole (40.2 % >0.5/9.5 µg ml^−1^) has been detected in carriage studies (D Lehmann, personal communication). Resistance of carriage isolates to other antimicrobials was consistent with isolates from children with meningitis (1996–2005); approximately 2.1 % of isolates from children with meningitis were tetracycline resistant and chloramphenicol and erythromycin resistance were rarely detected [2].

Colonisation with *S. pneumoniae* is a pre-requisite for invasive pneumococcal disease (IPD) [12]. Pneumococcal carriage is exceptionally high in PNG, with studies indicating all infants are colonised by 3 months of age and the median age of first pneumococcal acquisition is approximately 19 days [13, 14]. Consequently, *S. pneumoniae* is a common cause of bacterial meningitis amongst children in PNG [2, 15]. A broad range of serotypes have been reported in both carriage and IPD in PNG, consistent with other highly endemic settings [16–20]. Prior to introduction of 13-valent pneumococcal conjugate vaccine (PCV13), over 50 % of IPD cases amongst children in PNG were caused by non-PCV13 serotypes, limiting potential benefit of the vaccine [2, 18]. Interestingly, some serotypes rarely observed in other geographic regions have been common causes of IPD in PNG, such as serotypes 2 and 46 [15, 16]. In PNG, the high rates of pneumococcal carriage [13, 14] combined with a high proportion of disease caused by non-vaccine serotypes highlight the potential for replacement of PCV13 serotypes with non-vaccine serotypes [2].

A GAVI-funded, universal nationwide vaccination programme for infants using the PCV13 began in 2014. However, PCV13 (comprised of serotypes 1, 3, 4, 5, 6A, 6B, 7F, 9V, 14, 18C, 19A, 19F, 23F) was not widely administered in the Eastern Highlands Province (EHP), where the majority of aetiology studies have been conducted, until late 2015 and annual coverage estimates nationally have been estimated to be ≤35 % [21]. Encouragingly, PCV13 vaccination was associated with a 57.4 % reduction in hospitalisation of children due to pneumonia and a 28.7 % reduction of hypoxic pneumonia in PNG [22].

Building upon insights gained through phenotypic isolate characterisation, the present study examines whole genome sequence data for IPD isolates from children in PNG, providing a high-resolution view of the *S. pneumoniae* population structure. The diversity of lineages, serotypes and antimicrobial resistance traits are characterised, providing a baseline of *S. pneumoniae* diversity prior to the national PCV13 introduction.

## Methods

A collection of 174 *S. pneumoniae* isolates from children aged under 8-years-old with IPD in PNG between 1989 and 2014 were whole-genome sequenced using an Illumina HiSeq platform. The isolates originated from multiple formal studies and hospital surveillance (detailed in Table 1) conducted by the PNG Institute of Medical Research (PNGIMR) in collaboration with clinicians at the local hospitals. Isolates were freeze-dried for storage at the PNG Institute of Medial Research, and all viable isolates that were available were sequenced. The majority of isolates were from patients in the Asaro Valley area (which includes Goroka town, the provincial capital, located at 1600 m above-sea-level) and other areas within a 1 h drive of Goroka. The majority of isolates (77.6%, *n*=135) were from a study of the aetiology of suspected meningitis cases of children admitted to Goroka Base Hospital (previously named Goroka Hospital, then Goroka General Hospital and later as Goroka Regional Hospital). These specimens were collected as requested by paediatricians, without pre-defined selection criteria. Whilst most of the studies/surveillance aimed to investigate the aetiology of pneumonia and/or meningitis, in the study investigating the epidemiology of typhoid (Table 1), patients were enrolled if they had a history of fever for 3 days or more, and therefore cases of bacteraemia due to *S. pneumoniae* and other pathogens were also identified. Invasive isolates were included from a study conducted at Modilon Hospital, Madang Province on the north coast of PNG [23] and from surveillance of pneumonia at Tari Hospital in the remote Hela Province (previously part of Southern Highlands Province) (unpublished data), hospitalised children without antimicrobial treatment prior to blood culture were included. Whole genome sequencing and quality control of paired-end reads was conducted according to the Global Pneumococcal Sequencing (GPS) Project protocol [24].

**Table 1.** Summary of isolate origins: study, source, clinical manifestation

	Study description and reference(s)	no. of isolates	Years of study	% (no.) isolated from CSF§	% (no.) associated with				
					Meningitis	Pneumonia	Meningitis and pneumonia	Other manifestation	Unknown
i	Aetiology of suspected meningitis among children admitted to Goroka Base Hospital∗. [2, 19] Samples from children with meningitis, no additional predefined selection criteria	135	1989–1992 1997–2003	92.6 (*n*=125)	88.9 (*n*=120)	2.3 (*n*=3)	7.5 (*n*=11)	0	0.7% (*n*=1)
Ii	Aetiology study of pneumonia in children aged <5 years admitted to Tari Hospital, Hela Province (unpublished) Hospitalised children were included if antimicrobials were not started prior to blood culture	2	1990–1991	0	0	0	0	0	100 (*n*=2)
iii	Multicentre Young Infant Study (WHO) – investigation of severe infections in children aged <3 months attending Goroka Base Hospital∗ Outpatient Department [19] Children were included if illness began at home and rectal temperature >=35°C or <35.5°C or had a cough, high respiratory rate, difficulty breathing, convulsions, fever or difficult to wake.	9	1991–1993	11.1 (*n*=1)	22.2 (*n*=2)	44.4 (*n*=4)	0	22.2 (bacteraemia, *n*=2)	11.1 (*n*=1)
iv	Assessment of the epidemiology and transmission of *Salmonella typhi* in the Asaro Valley, EHP‡ (unpublished) Children with 3+ day history of fever resident in Asaro valley villages who attended the Goroka Base Hospital or Asaro Health Centre.	1	1993	0	0	0	0	0	100 (*n*=1)
v	Hospital-based invasive disease surveillance† (blood culture), Goroka Hospital∗, EHP‡ Samples from children with meningitis/sepsis with no additional predefined selection criteria	21	1995–1998, 2001	23.8 (*n*=5)	52.4 (*n*=11)	14.3 (*n*=3)	14.3 (*n*=3)	0	19.0 (*n*=4)
vi	Is routine lumbar puncture indicated in PNG child with febrile convulsion? (Modilon Hospital, Madang Province) [23, 45] Hospitalised children with one or more fever associated seizure	2	2007–2008	50 (*n*=1)	100 (*n*=2)	0	0	0	0
vii	Aetiology of moderate or severe pneumonia and meningitis in children aged <5 years enrolled through urban outpatient clinics or admitted to Eastern Highlands Provincial Hospital∗, Goroka, EHP‡ [25] Samples from children with pneumonia and meningitis, no additional predefined selection criteria	4	2013–2014	25 (*n*=1)	25 (*n*=1)	75 (*n*=3)	0	0	0

∗Goroka Regional Hospital previously known as Goroka General Hospital, Goroka Base Hospital, and Goroka Hospital; Goroka located in the Asaro Valley.

†Opportunistic sampling as requested by paediatrician.

‡EHP Eastern Highlands Province.

§Remainder of isolates were from blood samples.

Specimen collection, blood and CSF culture, phenotypic pneumococcal serotyping and antimicrobial susceptibility have been described in detail elsewhere [1, 2, 15, 19, 25]. Serotyping and antimicrobial susceptibility for all studies were conducted at the PNGIMR laboratory in Goroka.

*In silico* assignment of serotype was inferred using SeroBA [26] with additional screening of isolates predicted to be ‘untypable’ by SeroBA using the Centres for Disease Control and Prevention’s (CDC) in-house serotyping pipeline [27] and manual characterisation of the cps region. Multi-locus sequence typing (MLST) was performed using SRST2 [28]. Prediction of antimicrobial resistance phenotype from whole-genome sequencing (WGS) data was conducted using the CDC antimicrobial resistance pipeline [27, 29] which generates minimum inhibitory concentration (MIC) predictions, and classification of isolates as susceptible, intermediate or resistant based on Clinical and Laboratory Standards Institute (CLSI) guidelines [30], including separate MIC thresholds for meningitis infections. Multidrug resistance (MDR) was defined as *in silico* predicted resistance to three or more antimicrobial classes. Global Pneumococcal Sequencing Cluster (GPSC) assignments were determined using PopPUNK [31]. This method uses nucleotide variation across the whole-genome to cluster isolates, enabling definition of lineages. Lineages local to PNG were defined as GPSCs not identified in other geographic locations in the GPS database (November 2020) and/or MLST not within the PubMLST database (November 2020).

Reads were mapped to a reference *S. pneumoniae* (ATCC 700669, NCBI accession number FM211187) using BWA-MEM (v0.7.17) [32], followed by indel realignment using GATK (v3.7.0) [33], deduplication with Picard Mark Duplicates v1.127 (available at http://broadinstitute.github.io/picard/) and variant calling and consensus pseudosequence generation using samtools v1.6 [34] and bcftools v1.5. Regions of recombination were then identified and masked from the alignment using Gubbins v3.0 [35]. Single nucleotide polymorphism data were then extracted from the masked alignment and used to generate a maximum-likelihood phylogeny, using RAxML (v8.2.8) [36]. The metadata and analysis results are available to view interactively using Microreact (https://microreact.org/project/jhj4cLf93pvVQVp4hPN9Gq). Lineage-specific phylogenies were generated by mapping to a lineage-specific reference (GPSC96 accession ERR1214695, references for other GPSCs are detailed in supplementary data), then recombination was identified (same approach as previously described). Regions of recombination were masked prior to generation of the GPSC phylogenies using RAxML.

Fisher’s Exact test, implemented in R [37], was used to test for differences between PCV13 serotype and non-PCV13 serotype PNG isolates, including clinical presentation, sample source (CSF and blood) and antimicrobial resistance.

Concordance between phenotypic and genotypic AMR profiles was evaluated. Discordance of phenotypic and genotypic AMR was classified according to error type. Major errors occurred where phenotypically susceptible isolates were genotypically predicted to be resistant. Very major errors occurred where isolates were phenotypically resistant but genotypically predicted to be susceptible. Data were compared where phenotypic MIC data were available, with consistent MIC cut-offs (CLSI guidelines) for both the predicted and phenotypic methods. Categorical variables including clinical manifestation, serotype, vaccine status (PCV13 or non-vaccine serotype). Sample source and GPSCs were compared using Fisher’s Exact test with the significance level of *P*<0.05 using R (v 3.0.6).

## Results

### Sampling

Analyses in this study utilised 174 isolates from children with clinical disease attributed to *S. pneumoniae* collected between September 1989 and January 2014 in PNG, prior to the introduction of PCV13. Metadata are summarised in Table 1, stratified by origin study. The majority of isolates were derived from CSF samples (76.4 %, *n*=133), and the remainder were isolated from blood samples. Almost all samples were collected in the Eastern Highlands Province (97.7 %, *n*=170), with two isolates from each of Madang and Hela Provinces. The majority of isolates (90.2 %, *n*=157) were from children ≤2 years of age, 6.3 % (*n*=11) aged between 2 and 5 years, and the remaining 1.7 % (*n*=3) aged between five and 8 years. Patient age was unknown for 1.7 % (*n*=3) of isolates.

Of the 94.8 % (*n*=165) of isolates from patients with known clinical diagnosis, the majority were from children with a diagnosis of meningitis (82.4 %, *n*=136). A further 8.5 % (*n*=14) of isolates were from children with both meningitis and pneumonia, and 7.8 % of isolates (*n*=13) were from children with pneumonia as sole diagnosis.

### Serotypes

**Fig. 1.**
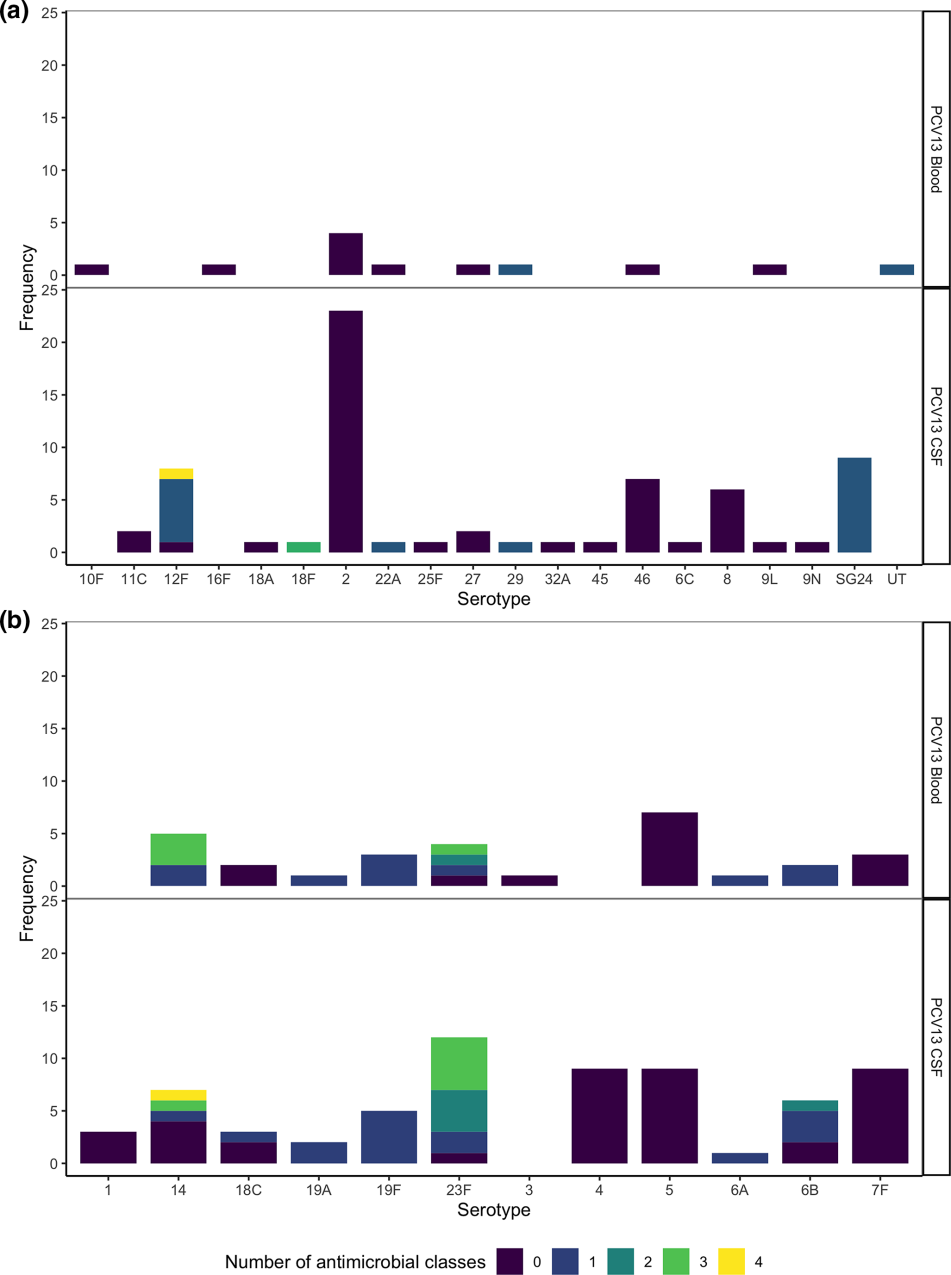
Summary of serotypes (≥3 isolates), grouped by sample source andpneumococcal conjugate vaccine (PCV) serotype status (part A non-vaccine serotype isolates; part B=PCV13 serotype isolates). The frequency bars are coloured by number of antimicrobial classes to which isolates were resistant. UT=untypable, SG24=serogroup 24. NVT=non-PCV13 serotypes. CSF=cerebrospinal fluid.

Almost half (45.4 %; *n*=79) of the total isolates were non-PCV13 serotype (NVT). The odds of CSF isolates expressing a non-PCV13 serotype was 2.44 times higher than for isolates from blood (95 % CI 1.10, 5.72; *P*=0.020). Overall, non-PCV13 serotype 2 was most common, followed by PCV13 serotypes 5, 23F, 7F and 14 (Fig. 1). Clinical manifestation data was available for 96.3 % (*n*=26) of serotype 2 isolates, of which most were from children with a diagnosis of meningitis alone (80.8 %; *n*=21), 11.5 % (*n*=3) pneumonia and meningitis and only 7.7 % (*n*=2) pneumonia alone. There was no significant association of clinical manifestation with serotype 2 isolates (*P*=0.871).

Compared with PCV13, the newly licensed PCV15 vaccine would not increase the proportion of vaccine type serotypes. A 10-valent PCV (Pneumosil), which includes serotypes 1, 5, 6A, 6B, 7F, 9V, 14, 19A, 19F, and 23F, would cover 72 (41.4 %) isolates and PCV20 (PCV13 plus serotypes 8, 10A, 11A, 12F, 15B, 22F, and 33F) would provide an incremental improvement in serotype coverage (58.1%, *n*=101). More encouragingly, a PCV24 vaccine (PCV13 serotypes plus serotypes 2, 8, 9N, 10A, 11A, 12F, 15B, 17F, 20, 22F, 33F), which is currently under development, would increase the proportion of vaccine serotype isolates in this study to 74.1 % (*n*=129). However, of the isolates expressing non-PCV24 serotypes 48.9 % (*n*=22) were predicted to be resistant to one or more antimicrobial.

One isolate was characterised as untypable using SeroBA and the CDC in-house serotyping pipeline; therefore, manual assessment of the cps region between dexB and aliA was conducted. cps genes were absent from the GPSC18 isolate, with only ΔIS1202Δ and pspKgenes between dexB and aliA, predictive of an unencapsulated phenotype. This isolate was also phenotypically untypable.

### GPSCs

Amongst the 174 isolates, 45 GPSCs were identified, of which 15 GPSCs were only detected in PNG within the GPS collection highlighted in Fig. S1 (available in the online version of this article). The STs of isolates within these GPSCs were searched against the PubMLST database, as a proxy for GPSC, and were not identified in other geographic regions. Interestingly, 28.4 % of isolates (*n*=50) belonging to 15 GPSCs were of a GPSC not identified in other geographic regions (GPSCs 635 (*n*=12), 595 (*n*=9), 640 (*n*=8), 656 (*n*=4), 658 (*n*=4); the remaining GPSCs were represented by ≤2 isolates).

The top five most common GPSCs were 96, 8, 635, 15 and 20 (Table 2). Of the 32 serotypes, 12 were expressed across multiple GPSCs (maximum five GPSCs). For almost all GPSCs there was a single expressed serotype Fig. S2. The exceptions were GPSC149 (serotypes 27 (*n*=2) and 32A (*n*=1)), GPSC635 (serotypes 19F (*n*=7), 14 (*n*=3), 19A (*n*=1), 29 (*n*=1)) and 713 (serotypes 6B (*n*=1), 6C (*n*=1)).

**Table 2.** Summary of isolate characteristics for GPSCs with >5 isolates, ranked by number of isolates

GPSC	Total no. of isolates	no. of isolates from CSF†	Years	Serotype(s) (*in silico* prediction)	Multilocus sequence type(s)	Clonal complex (CC)	Antmicrobial resistance (*in silico* prediction)
96	27	23	1989–2002	2	1504 (*n*=24) 16705 (*n*=3)	74	None
8	16	9	1990–2002	5	16188∗	289	None
15	11	9	1991–2003	7F	191 (*n*=6) 16198∗ (*n*=5)	191	None
635∗	12	5	1991–1997	19F (*n*=7) 14 (*n*=3) 19A (*n*=1) 29 (*n*=1)	16204 (*n*=5) 6904 (*n*=2) 16210 (*n*=2) 16244 (*n*=2) 16068 (*n*=1)	CC63 (*n*=1) CC unassigned (*n*=11)	Resistant to penicillin (meningitis MIC threshold)
20	10	8	1990–2003	23F	802 (*n*=8) 16189∗ (*n*=2)	802 (*n*=8) CC unassigned (*n*=2)	ST802 isolates: intermediatesusceptibility cotrimoxazole (*n*=8) and resistant to tetracycline (*n*=6) ST16189: none
162	8	8	1990–2001	4	14136 (*n*=7) 4127 (*n*=1)	4127	None
595∗	9	9	1990–2002	Serogroup 24	6755	CC unassigned	Resistant to penicillin at meningitis MIC thresholds
334	7	7	1990–2000	12F	16297∗ (*n*=6) 1527 (*n*=1)	1527 (*n*=1) CC unassigned (*n*=6)	Isolates from 1991 onward (*n*=6) resistant to penicillin at meningitis MIC threshold. Isolates from 1990: none
640∗	8	7	1995–2002	46	16207∗ (*n*=6) 16246∗ (*n*=2)	CC unassigned	None
5	5	4	1990–2002	23F	16193	172	Resistant to penicillin at meningitis threshold and resistant to cotrimoxazole (*n*=5)
76	6	4	1998–2014	6B	16206 (*n*=5) 16259 (*n*=1)	CC unassigned	Resistant to penicillin at meningitis threshold. One ST16206 isolate resistant to cotrimoxazole
224	6	6	1991–2003	8	16229∗ (*n*=1) 6748∗ (*n*=5)	6022	None

*Not described outside Papua New Guinea (within GPS and PubMLST databases). Data for the remaining 33 GPSCs in supplementary data, antimicrobial susceptibility characteristics summarised in Fig. S1 and serotypes summarised in Fig. S2.

†Remainder of isolates derived from blood samples.

**Fig. 2.**
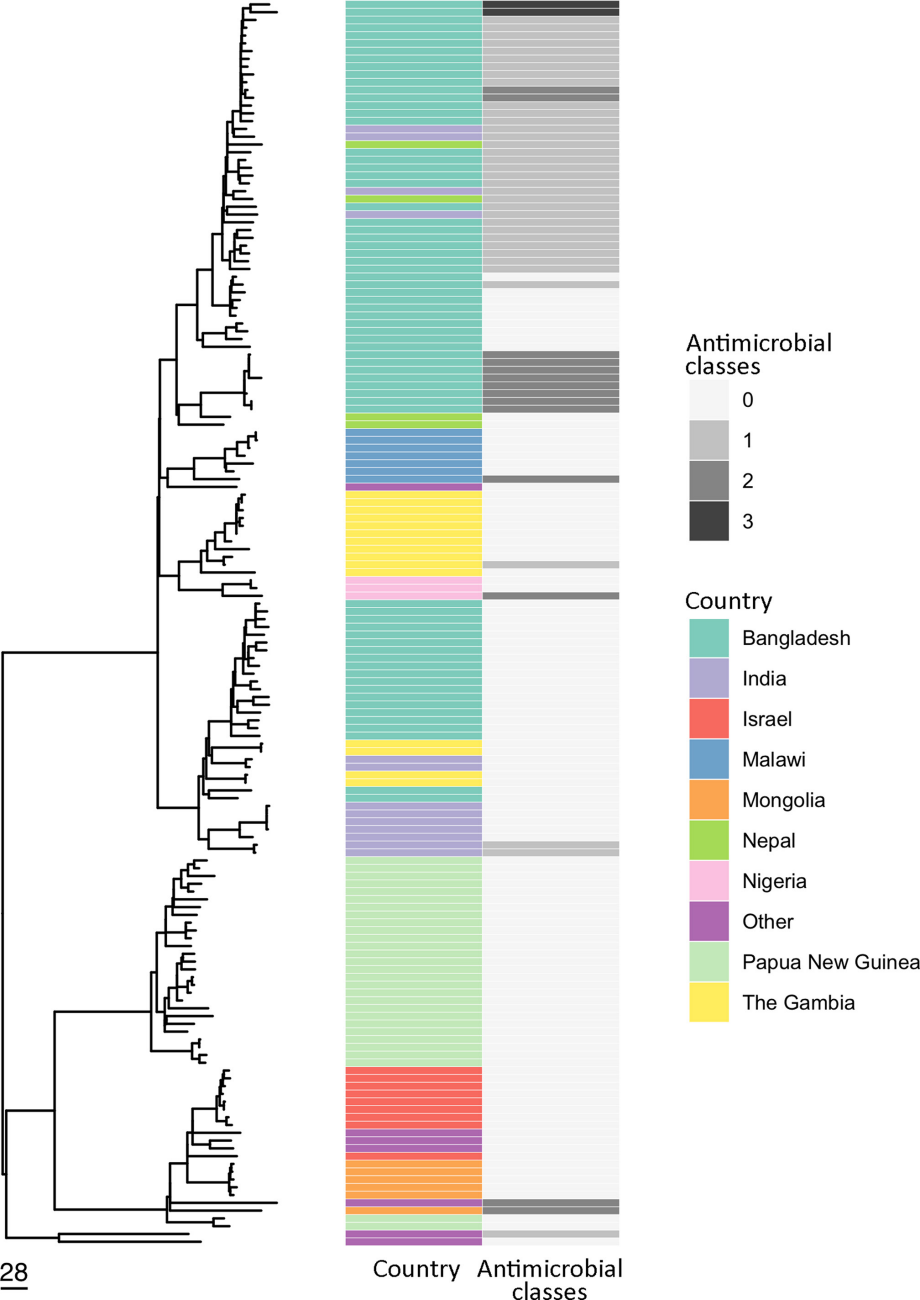
Phylogeny of Papua New Guinea and published Global Pneumococcal Sequencing collection serotype 2, GPSC 96 isolates. Heat map displays the country of isolate origin and number of antimicrobial classes to which the isolates were predicted to be resistant (using the CDC pipeline). Refererence mapped phylogeny (reference: ERR1214695), post-masking of recombination sites using Gubbins and generated using RAxML.

Phylogenies were constructed for GPSCs with >5 isolates from this study, placing isolates from PNG in the context of global GPS collection isolates (Fig. S3). GPSC96 isolates were all serotype 2, the most common serotype in the collection, and formed two distinct phylogenetic clusters which were concordant with MLST designations. The majority (*n*=27) of GPSC96 isolates, spanning 1998–2002, clustered together and were distinct from other GPS collection isolates. Two GPSC96 isolates, from 2000 and 2001, formed a distinct cluster and were most closely related to isolates from Israel (average 82 SNPs) and Morocco (average 86 SNPs) (Fig. 2). Similarly, all GPSC20 isolates were serotype 23F, and formed two distinct clusters within phylogenies of isolates from diverse geographic regions, consistent with MLSTs and AMR profiles. In contrast, within GPSCs 15, 76, 224 and 334 the PNG isolates formed a single, distinct cluster within each global GPSC phylogeny (Fig. S3).

### Antimicrobial resistance

Over a third of isolates (36.2%, *n*=63) were predicted to be resistant to one or more antimicrobial and 7.3 % (*n*=14) multidrug resistant. For six GPSCs (20, 334, 40, 658, 76) more than one AMR profile was observed. Multidrug resistant isolates included all GPSC658 (*n*=4, serotype 14), GPSC26 (*n*=1, serotype 12F) and 60 % (*n*=6) of GPSC20 (serotype 23F) isolates. No isolates collected prior to 1998 were classified as MDR. PCV13 serotype isolates had 10.1 times greater odds of being MDR than non-PCV13-serotype isolates (95 % CI [1.41, 444.3], *P*=0.007). No isolates from GPSCs exclusive to PNG were predicted to be MDR.

At the MIC threshold for meningitis (0.12 µg ml^−1^), 24.1 % of NVT serotype isolates and 32.6 % of PCV13 isolates were classified as resistant to penicillin, with no significant difference in odds of penicillin resistance between NVT and PCV13 serotype isolates (OR 0.66 [0.31, 1.34], *P*=0.241). Most NVT penicillin-resistant strains were serogroup 24 (*n*=9, GPSC595), 19F (*n*=8, GPSC635) or 12F (*n*=6, GPSC334); other serotypes included 29 (*n*=2) and 22A (*n*=1). PCV13 serotype penicillin-resistant strains were 14 (*n*=8), 23F (*n*=6), 6A (*n*=2), 19A (*n*=1). Interestingly, GPSC334 isolates (*n*=6) from 1991 onwards were predicted to be resistant to penicillin which appears to be due to recombination events of both *pbp2B* and *pbp2X*, resulting in import of novel *pbp2B* and *pbp2X* alleles, while the GPSC334 isolate from 1990 was predicted to be susceptible to antimicrobials. Amongst the 50 isolates predicted to be resistant to penicillin at the meningitis MIC threshold, resistance was encoded by seven unique allelic profiles of *pbp1A, pbp2B* and *pbp2X* genes, of which six profiles had one or more ‘new’ alleles. An allelic profile containing one or two ‘new’ *pbp* alleles was observed in 12 isolates, each unique to a GPSC. A profile of three novel alleles predicted to confer resistance to penicillin was common to 33 isolates across ten GPSCs. Of the isolates with phenotypic penicillin MIC data (96.6%, *n*=168), there was 93.5 % concordance between phenotypic and genotypic predicted penicillin resistance at the meningitis MIC threshold. Discordance comprised of very major errors for two isolates and major errors for nine isolates.

Cephalosporin-resistant isolates (*n*=4) were all GPSC 658, serotype 14 isolates; these isolates were also cotrimoxazole- and penicillin-resistant (Fig. S1). Cotrimoxazole resistance, encoded by indels in *folP* (five variants) with or without point mutations in *folA*, was predicted for 5.7 % (*n*=10) of isolates and a further 7.5 % (*n*=13) of isolates were predicted to have intermediate susceptibility to cotrimoxazole. Of the cotrimoxazole-resistant isolates, ten expressed PCV13 serotypes (GPSC5, serotype 23F (*n*=5); GPSC658, serotype 14 (*n*=4)); GPSC76, serotype 6B (*n*=1). There was concordance between phenotypic and genotypic predicted cotrimoxazole resistance for 95.8 % (*n*=159) of the 166 isolates with available phenotypic MIC data. Tetracycline-resistant isolates were detected from 1998 onwards, encoded by *tet*(M) in 5.2 % of isolates (*n*=9) across both PCV13 serotype isolates (GPSC 9, serotype 14 (*n*=1); GPSC 20, serotype 23F (*n*=6)) and non-PCV13 serotype isolates (GPSC26, serotype 12F (*n*=1); GPSC172, serotype 18F (*n*=1)). For the 60 isolates with phenotypic tetracycline MIC data there was 96.7 % concordance between phenotype and genotype.

## Discussion

Here we show high serotype and lineage diversity amongst IPD *S. pneumoniae* isolates from PNG. A distinct *S. pneumoniae* population compared to other sampled geographic locations was evident; multiple common *S. pneumoniae* lineages were observed across extended time periods between 1990 and 2014 in PNG and were not observed in other countries based on the GPS or PubMLST databases. The most common serotypes associated with IPD cases in PNG appear to differ from those observed in carriage in neighbouring Indonesia, where serogroups 6 and 23, and untypable isolates have been the most commonly isolated [38–40]. These findings are indicative of diverse serotypes circulating in PNG for an extended time period and limited long-range transmission events to or from PNG; however, a lack of representation of isolates from neighbouring countries limit the assessment of lineage distributions.

The most common predicted antimicrobial resistance trait was resistance to penicillins at the meningitis MIC threshold, including amongst GPSC 635, 640 and 595 isolates which were exclusive to PNG. As there was no significant difference in resistance to penicillins between NVT and PCV13 serotype isolates, the introduction of PCV13 would not be anticipated to reduce proportionate penicillin resistance of *S. pneumoniae* meningitis cases. However, as PCV13 serotype isolates had higher odds of being MDR compared with NVT serotypes, a reduction in MDR strains may occur, though in the future could be anticipated to return to a similar level due to ongoing selection [41].

Almost half of isolates expressed non-PCV13 serotypes, reflective of previous reports from PNG [2; Lehmann *et al*., 1999 [18, 20]. This contrasts with estimates that PCV13 would cover 74 % of IPD in all geographic regions globally, including Oceania [17]. Two GPSCs (635 and 713) expressed both PCV13 and NVT serotypes, suggesting potential for serotype replacement by these lineages. Interestingly, one unencapsulated GPSC18 isolate was identified from a blood sample from a child with meningitis. This strain would not be expected to have been the causative agent of meningitis and the possibility of a mixed pneumococcal infection cannot be excluded [42]. All GPSC635 were penicillin-resistant and all GPSC5 isolates were resistant to both cotrimoxazole and penicillin, reducing treatment options.

GPSC96 was the dominant GPSC represented amongst the PNG dataset utilised for this study. Concerningly, serotype 2 is not included in the PCV13 currently used in PNG, though is included in the higher valency PCV24 Affinivax vaccine. The GPSC96 isolates were all serotype 2, which has been associated with IPD in other geographic regions including Bangladesh and Israel [43, 44]; and reported in PNG for over 30 years [15, 16]. In PNG, the distribution of serotype 2 across age groups has appeared to be similar to other serotypes [2]. However, the epidemiology of serotype 2 can vary between geographic settings; in Bangladesh serotype 2 has been overrepresented, relative to other serotypes, amongst young children [44]. The association of serotype 2 with clinical manifestation also appears to vary with location, though may be influenced by sampling; in Israel the most common presentation was pneumonia (78.5%) followed by septicaemia (9.9%) and meningitis (7.4%) [43]. In stark contrast, in Bangladesh 98 % of serotype 2 IPD cases had a diagnosis of meningitis [44]. In PNG serotype 2 *S *. *pneumoniae* were isolated from children with meningitis and those with pneumonia alone, with no significant association with clinical manifestation. However, sample numbers were limited and cases may not be reflective of the wider population. The global GPSC96 phylogeny revealed two clusters of PNG isolates, with a main clonal cluster comprising of isolates spanning 1989 to 2002 which may be representative of long-term serotype 2 isolates circulating in PNG. These isolates were distantly related to GPSC96 isolates from other geographic regions. In contrast, the cluster of two PNG GPSC96 isolates most closely related to isolates from Israel and Morocco may indicate a long-range transmission event to or from PNG.

The samples available for this study derived from both routine surveillance and multiple studies in PNG, pooled to provide an overview of genomic characteristics of isolates from a location with limited available data. The *S. pneumoniae* captured by this overview will not be fully representative of IPD cases in PNG, however, in the absence of systematically derived surveillance data, the isolates contributing to this study provide a valuable baseline for the evaluation of the impact of PCV13 upon the *S. pneumoniae* population causing IPD in children in the EHP as the majority of isolates in this study were from this province. The pneumococcal population structure observed in this study may not be representative of other regions of PNG, and comparisons of *S. pneumoniae* pre- and post-perturbation due to PCV13 introduction may therefore be limited to EHP. Additionally, the majority of isolates were from patients with meningitis rather than pneumonia, which is not reflective of hospitalised case numbers [7]. Expansion of surveillance to capture *S. pneumoniae* in carriage, and causing both meningitis and pneumonia across PNG is needed to provide a robust basis for the assessment of the impact of vaccine introductions.

The resolution afforded by genomic data and contextualisation using the GPS collection has highlighted the distinct *S. pneumoniae* population observed to cause IPD in children in PNG. Ongoing standardized surveillance, including WGS of isolates, post-PCV13 distribution is essential to evaluate serotype replacement patterns and impact of PCV13 upon AMR traits. Additionally, surveillance needs to be conducted at several representative sites across the country, perhaps using sampling from healthy carriage alongside pneumococcal disease.

## Supplementary Data

Supplementary material 1

Supplementary material 2
