# Supplementary material for: Distinct Streptococcus pneumoniae cause invasive disease in Papua New Guinea
Source: Microb Genom. 2022 Jul 11;8(7):mgen000835. doi: 10.1099/mgen.0.000835 (PMC9455700; doi:10.1099/mgen.0.000835)
Supplement: Supplementary material 1 [file mgen-8-835-s001.pdf]

Supplementary Figures

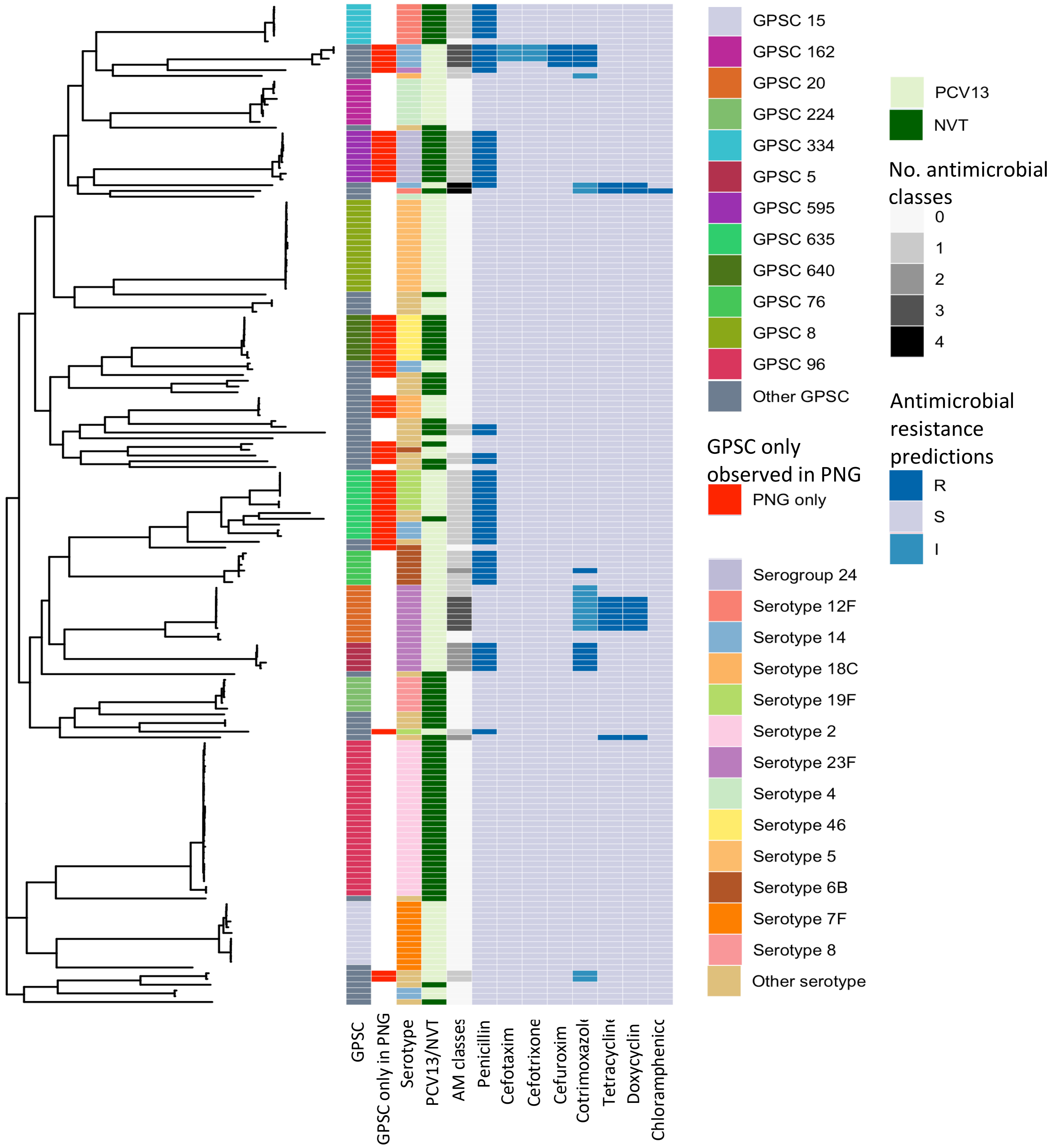

Supplementary Figure 1. Phylogenetic tree of PNG isolates with heatmap of *in silico* antimicrobial resistance predictions, and serotypes. GPSCs and serotypes observed 5 or fewer times comprise the 'Other' category. GPSCs observed in PNG but not in other countries in the Global Pneumococcal Sequencing project dataset are highlighted in the 'GPSC only in PNG' heatmap column.

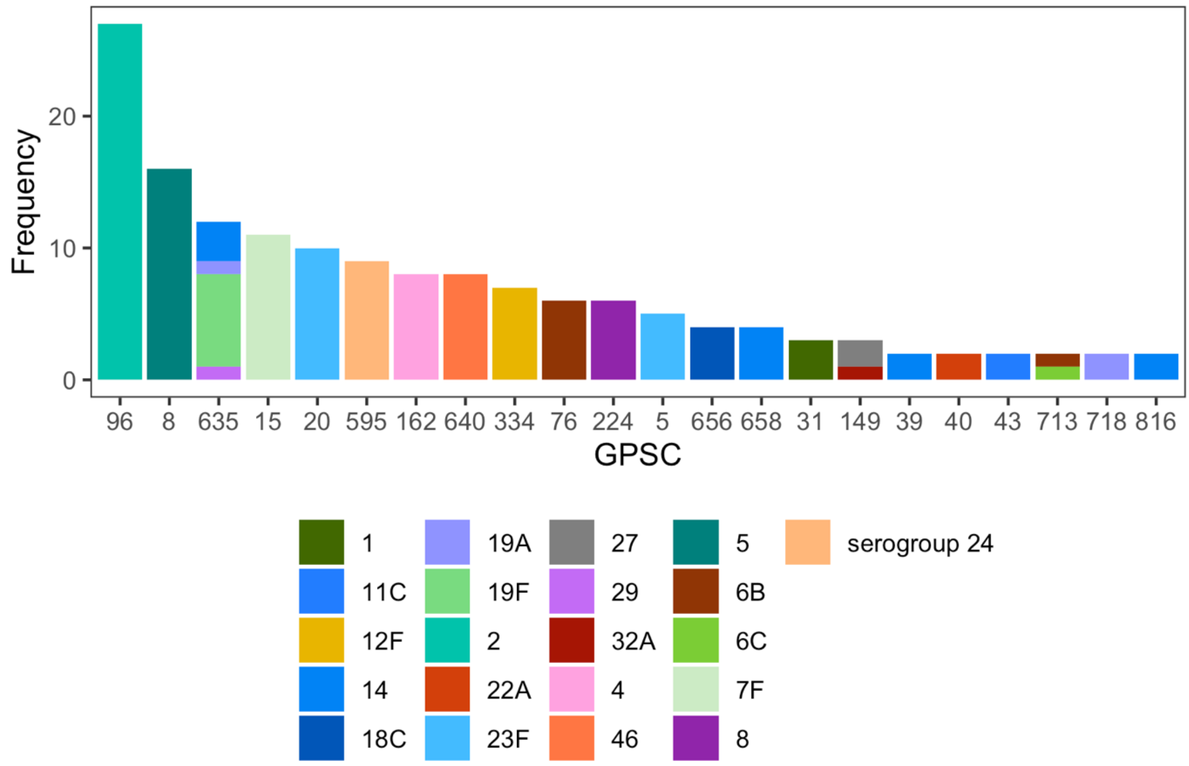

Supplementary Figure 2. GPSCs rank ordered by isolate count and categorized by serotype (data shown where >1 isolate in GPSC)

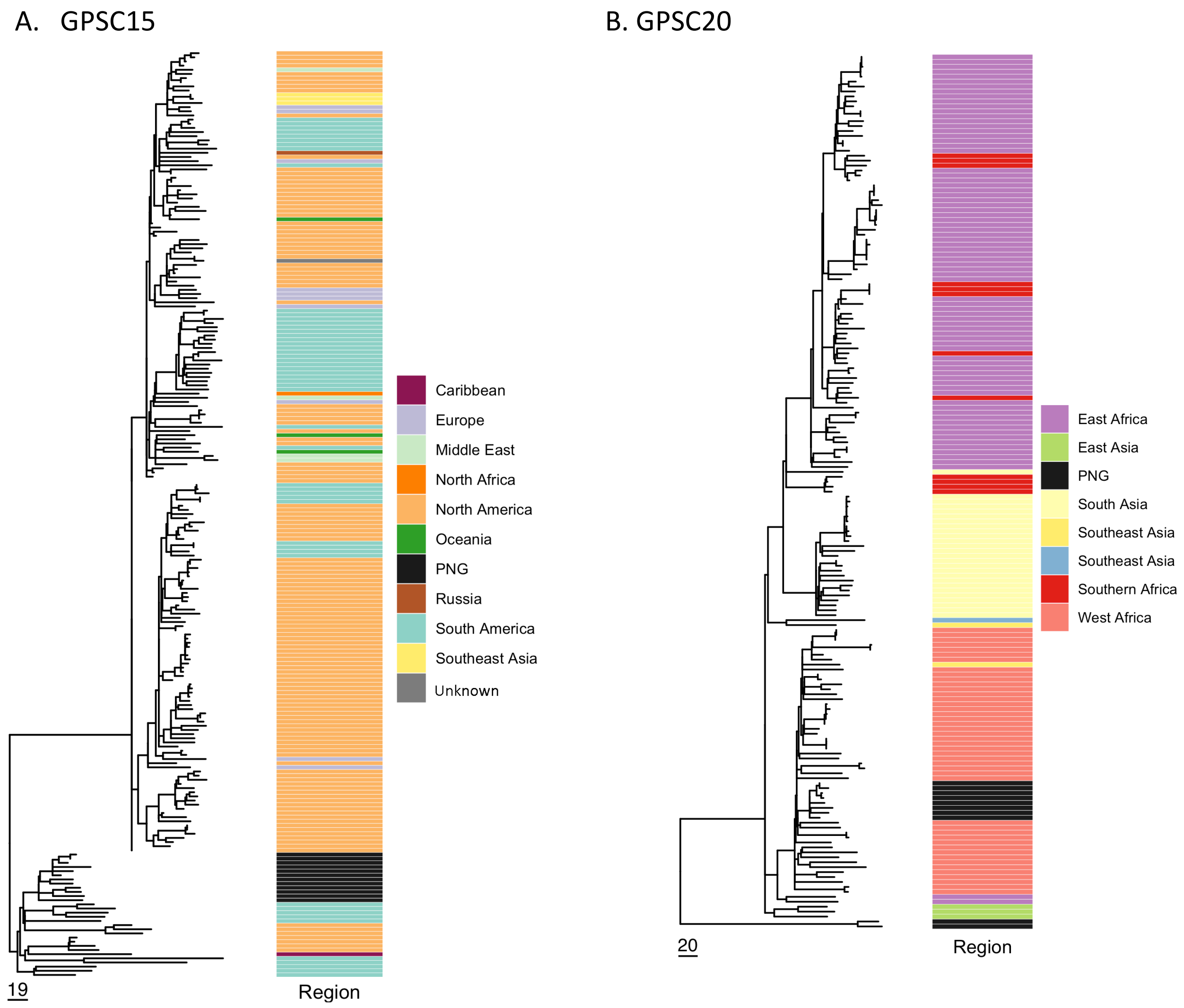
A. GPSC15
19
Region
Caribbean
Europe
Middle East
North Africa
North America
Oceania
PNG
Russia
South America
Southeast Asia
Unknown
B. GPSC20
20
Region
East Africa
East Asia
PNG
South Asia
Southeast Asia
Southeast Asia
Southern Africa
West Africa

C. GPSC76

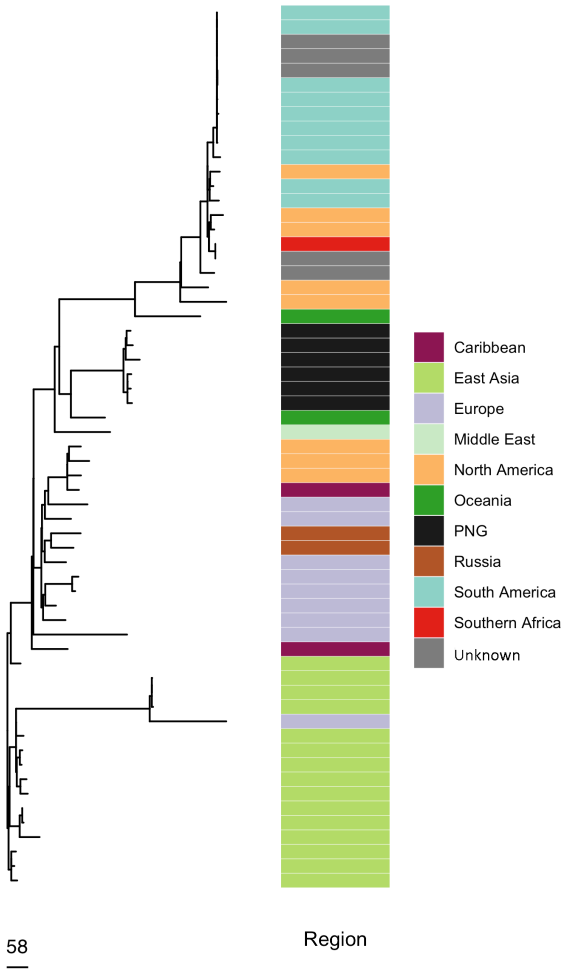

D. GPSC162

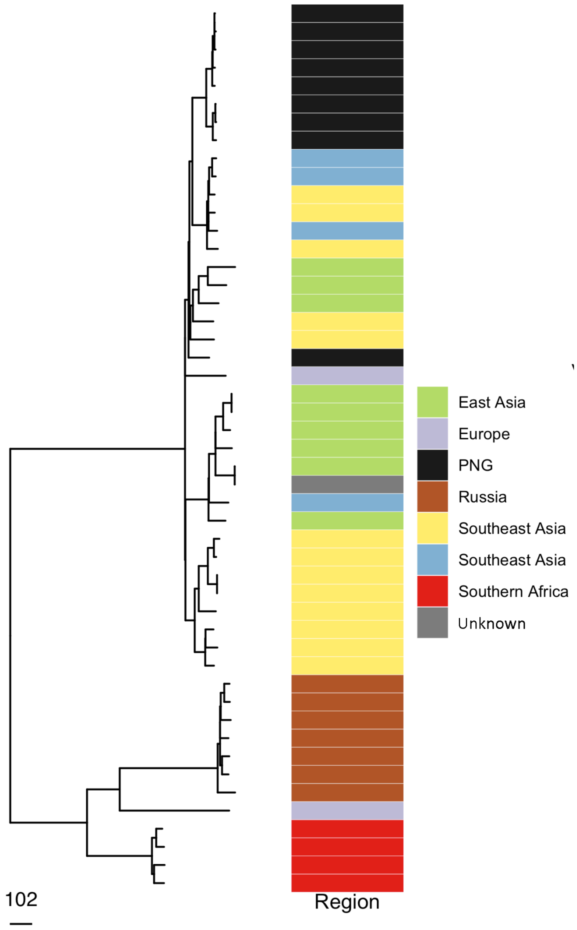

E. GPSC224

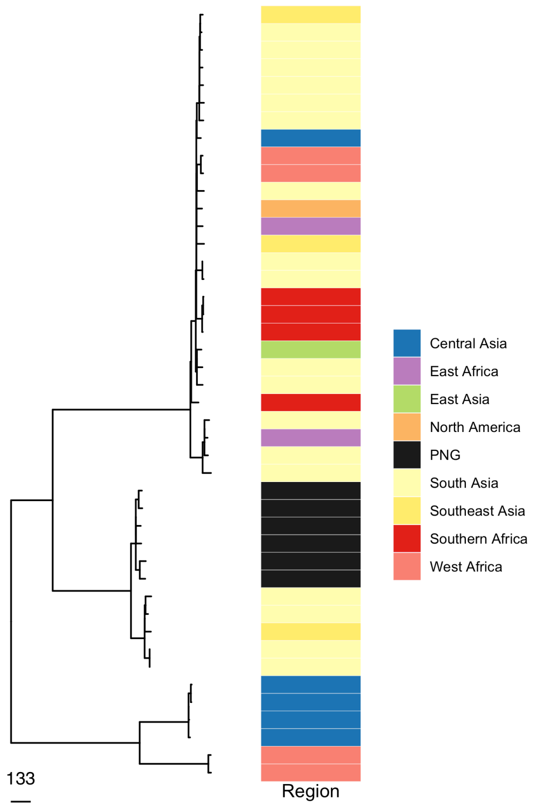

F. GPSC334

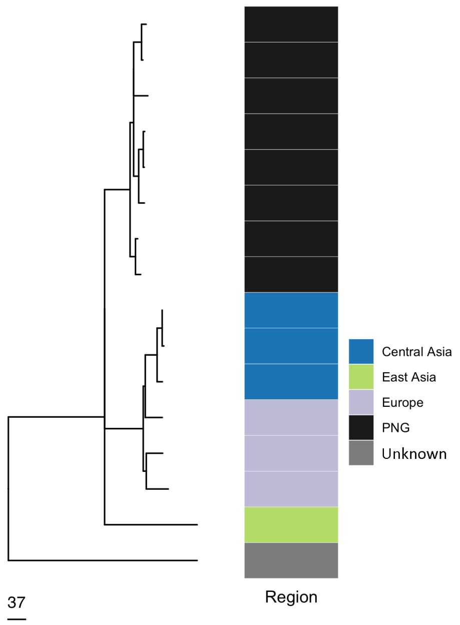

Supplementary Figure 3. Phylogenies of Papua New Guinea and published Global Pneumococcal Sequencing collection isolates for individual GPSCs. The heatmaps display the region of isolate origin. The alignments used for phylogeny generation were created through mapping of reads to the lineage specific reference for each GPSC. GPSC15 reference: ERR449143. GPSC20 reference: ERR913259. GPSC76 reference: ERR449183. GPSC162 ERR2366798. GPSC224 reference: ERR3270757. GPSC334 reference: NZ_AP019192. Scale bar = number of SNPs.
